# Biodiversity Increases the Productivity and Stability of Phytoplankton Communities

**DOI:** 10.1371/journal.pone.0049397

**Published:** 2012-11-16

**Authors:** Alina A. Corcoran, Wiebke J. Boeing

**Affiliations:** Department of Fish, Wildlife and Conservation Ecology, New Mexico State University, Las Cruces, New Mexico, United States of America; University of Zurich, Switzerland

## Abstract

Global biodiversity losses provide an immediate impetus to elucidate the relationships between biodiversity, productivity and stability. In this study, we quantified the effects of species richness and species combination on the productivity and stability of phytoplankton communities subject to predation by a single rotifer species. We also tested one mechanism of the insurance hypothesis: whether large, slow-growing, potentially-defended cells would compensate for the loss of small, fast-growing, poorly-defended cells after predation. There were significant effects of species richness and species combination on the productivity, relative yield, and stability of phytoplankton cultures, but the relative importance of species richness and combination varied with the response variables. Species combination drove patterns of productivity, whereas species richness was more important for stability. Polycultures containing the most productive single species, *Dunaliella*, were consistently the most productive. Yet, the most species rich cultures were the most stable, having low temporal variability in measures of biomass. Polycultures recovered from short-term negative grazing effects, but this recovery was not due to the compensation of large, slow-growing cells for the loss of small, fast-growing cells. Instead, polyculture recovery was the result of reduced rotifer grazing rates and persisting small species within the polycultures. Therefore, although an insurance effect in polycultures was found, this effect was indirect and unrelated to grazing tolerance. We hypothesize that diverse phytoplankton assemblages interfered with efficient rotifer grazing and that this “interference effect” facilitated the recovery of the most productive species, *Dunaliella*. In summary, we demonstrate that both species composition and species richness are important in driving patterns of productivity and stability, respectively, and that stability in biodiverse communities can result from an alteration in consumer functioning. Our findings underscore the importance of predator-prey dynamics in determining the relationships between biodiversity, productivity and stability in producer communities.

## Introduction

With unprecedented species extinction rates [1], [2], [3] and concomitant changes in ecosystem functioning [4], [5], [6] worldwide, there is a need to understand the relationships between biodiversity, productivity and stability at different trophic levels. Although there is no simple relationship between productivity and species richness [7], it is generally accepted that productivity and stability increase with biodiversity at the community level [8], [9], [10], [11], [12]. One mechanism that has been proposed to explain positive biodiversity-productivity relationships is niche partitioning, whereby species with different morphological or physiological characteristics can use different resources thus increasing overall productivity in species rich systems [13], [14], [15], [16]. Extending this concept from productivity to stability, a functionally diverse group of taxa may be more resistant or resilient to environmental or biological perturbations because different species exhibit different tolerances and thus responses to changes in environmental and biological factors. This idea, termed the “insurance” hypothesis or effect [17], [18], [19], has received some strong [10] but not unequivocal support [9], [20], [21].

In phytoplankton communities, functional diversity can arise when species exhibit different traits along a few key axes, namely light or nutrient utilization and susceptibility to predators. Major taxonomic groups (e.g. diatoms, dinoflagellates, cyanobacteria) tend to differ in their mean functional trait values, despite physiological or morphological plasticity within groups [22]. It follows that communities with representative species from diverse taxonomic groups may be more productive or stable than communities with species from fewer groups. Indeed, experimental manipulations [23], as well as analyses of field-collected data [24], [25], support niche partitioning as a mechanism that underlies positive biodiversity-productivity relationships in phytoplankton communities. Yet, there has not been consistently strong support for positive biodiversity-productivity relationships in experimental phytoplankton communities [21], [26]. Furthermore, little attention has been devoted to understanding stability resulting from functional traits within phytoplankton communities.

Here we describe the relationships between biodiversity, productivity and stability within phytoplankton communities, and we test the insurance hypothesis in the context of grazing tolerances. Specifically, we test the hypothesis that grazing-tolerant cells will compensate for the predatory loss of poorly-defended cells, resulting in greater productivity and stability, defined temporally, of polycultures compared to monocultures. There are two novel aspects to this study. First, to study stability we impose a biological perturbation. Previous studies that investigated stability of phytoplankton communities used environmental perturbations [21], [27]. The outcomes of biological perturbations, likely more complex than environmental perturbations, have particular relevance to understanding natural populations that are also subject to changing pressures from predation, disease, competition, immigration, or emigration. Second, we test compensatory dynamics explicitly by creating phytoplankton polycultures with species that are likely to differ in their ability to resist predation. With this design, we show that algal polycultures are more productive and stable than algal monocultures, due to indirect predator-mediated effects. This finding highlights the importance of predator-prey dynamics in understanding the role of biodiversity in producer communities.

## Methods

### Study Organisms and Culture Conditions

Our study organisms included six phytoplankton species that were grouped into two functional groups according to growth rate and susceptibility to grazing (Table 1) by *Brachionus plicatilis*, a brachionid rotifer that suspension feeds by drawing particles into its corona with ciliated buccal fields. Relative phytoplankton growth rate was measured directly with laboratory cultures, and grazing tolerance was assessed based on morphology. We considered the spines of *Chaetoceros*, chain-formation of *Chaetoceros* and *Melosira*, size of *Coscinodiscus*, and silica frustules of all three larger species (Table 1) to be morphological defenses that would deter or prohibit grazing by the rotifer. Although *B. plicatilis* is capable of consuming particles up to 58 µm, siliceous frustules are thought to be effective defenses against predators for diatoms [28], [29], [30]. All species except *Nannochloris oculata*, which was already in culture, were obtained from the National Center for Marine Algae and Microbiota (formerly the Culture Center for Marine Phytoplankton, Bigelow, ME). Cultures were acclimated through consecutive batch transfers to one media type (f/2 with artificial seawater [31], [32], [33]), temperature (19°C) and light cycle (16 h light:8 h dark by cool white fluorescent lights that supplied 300 µmol photons m^−2^ s^−1^). Before starting the experiment, cultures were maintained in 250 mL flasks for approximately two months in a single incubator (I-36VL, Percival Inc., Perry IA). Although phytoplankton cultures were not axenic when the experiment was started, bacterial abundance in the non-axenic cultures was low (<1% of total biomass).

**Table 1:** Classification and key morphological characteristics of the study species.

Species	ID #	Division	Class	Size	Key Characteristics
fast-growing, susceptible to grazing					
*Nannochloris oculata*	––	Chlorophyta	Chlorophyceae	2–3 µm	non-motile, green to yellow
*Dunalliela tertiolecta*	1320	Chlorophyta	Chlorophyceae	6–11 µm	biflagellate, green pigments at posterior
*Rhodomonas* sp.	768	Cryptophyta	Cryptophyceae	11–16 µm	biflagellate, red to light brown
slow-growing, potentially defended against grazers					
*Chaetoceros decipiens*	173	Heterokontophyta	Bacillariophyceae	10–26 µm	straight chains, long setae, yellow to brown
*Melosira octogona*_cf	483	Heterokontophyta	Coscinodiscophyceae	19–25 µm	cylindrical cells, chain-forming, yellow to brown
*Coscinodiscus* sp.	1583	Heterokontophyta	Coscinodiscophyceae	75–90 µm	solitary, thick frustules, yellow to brown

Species were divided into two functional groups, as indicated. Identification numbers from the National Center for Marine Algae and Microbiota (formerly the CCMP) are listed. Measurements of cell length and width or diameter and height were made on at least 25 cells per species. Size ranges encompass all dimensions.

### Experimental Design

The experimental design included three richness treatments (two, four and six species), within which species combinations were nested (Table 2), plus monoculture controls. Species combinations were created by haphazardly selecting species from each of the two functional groups such that functional groups were equally represented in all treatments. Experimental cultures were inoculated using a replacement design, with the initial cell abundance constant across levels of richness and species combinations. This experiment was a follow-up experiment to one conducted in which starting biovolume was constant. As both experiments yielded similar results, we do not present our preliminary data here. Our design resulted in equal average starting biovolume (1.37 × 10^7^ µm^3^/mL) across richness treatments because species occurred in the experimental design an equal number of times (Table 2). However, biovolume varied between species combinations, from 1.4 × 10^5^ µm^3^/mL in the *Chaetoceros* and *Nannochloris* treatment to 4 × 10^7^ µm^3^/mL in the *Coscinodiscus* and *Dunaliella* treatment. As such, initial biovolume was used as a covariate in statistical analyses (see “Data Analyses”). Inoculum was added to 20 mL glass tubes (16 mm outer diameter) with 15 mL f/2 media, resulting in an initial cell abundance of 170 cells/mL. All tubes were placed in an angled tissue culture roller drum (TC-7, New Brunswick Scientific, Edison NJ), which rotated at 15 rpm, in the same incubator in which stock cultures were maintained. Before sampling, cultures were grown for approximately two weeks to allow communities to assemble and populations to reach measurable abundance.

**Table 2:** Experimental design of richness and species combination treatments, with the number of replicates as indicated.

Richness	Species Combinations
	(a) *Chaetoceros* & *Nannochloris* [n = 3]
2 [n = 9]	(b) *Melosira* & *Rhodomonas* [n = 3]
	(c) *Coscinodiscus* & *Dunaliella* [n = 3]
	(d) *Chaetoceros, Melosira, Nannochloris* & *Rhodomonas* [n = 3]
4 [n = 9]	(e) *Coscinodiscus, Dunaliella, Melosira* & *Nannochloris* [n = 3]
	(f) *Chaetoceros, Coscinodiscus, Dunaliella* & *Rhodomonas* [n = 3]
6 [n = 9]	(g) all species combined [n = 9]Each monoculture control was grown in triplicate.

After the two-week growth period, we measured net community production and sampled cultures to estimate total community biovolume every four days for 12 days (see “Response Variables”). Immediately after the third sampling point, we added 15 rotifers (L-type, *B. plicatilis*, Reed Mariculture, Campbell CA) to each culture tube. Before this predator addition, the rotifers were starved for a day and rinsed in sterile seawater to reduce the introduction of bacteria to the phytoplankton cultures. After rotifer addition, cultures were sampled for the aforementioned variables, as well as rotifer abundance, every four days for an additional 12 days.

### Response Variables

Net community production was determined by direct measurement of O_2_ evolution in the headspace of culture vessels [34]. Prior to incubations, cultures were bubbled with the reference gas for two minutes to prevent carbon limitation during the incubation period and establish a baseline O_2_ concentration. After bubbling, cultures were incubated for approximately four hours and final concentrations of O_2_ in the headspace of the tubes were measured by flushing air from the headspace into an Oxzilla II Differential Oxygen Analyzer (Sable Systems, Henderson, NV) during a three-minute sampling period. After measuring O_2_ production, one milliliter (<7% of culture volume) of each culture was preserved with Lugol’s Iodine, and fresh media was used to replace the sampled volume. Preserved cells were enumerated under light and phase contrast microscopy (BH-2, Olympus, Central Valley PA). At least 400 cells and comparable numbers of each species per sample were counted (e.g. 200, 100, and 70 cells of each species in the two-, four-, and six-species combinations, respectively). *Chaetoceros*, *Rhodomonas* and *Melosira* were counted at 200X and *Coscinodiscus* at 100X using a gridded Sedgewick-Rafter chamber (1801–G20, Wildlife Supply Company, Yulee FL). *Dunaliella* and *Nannochloris* were counted in a Neubauer hemocytometer (Marienfeld GMBH & Co., Germany) at 200X and 400X, respectively. To estimate total biovolume, species abundance was multiplied by mean biovolume, calculated before the experiment using measurements of>25 cells per species and formulas of Hillebrand et al. [35]. Measurements made at the end of the experiment confirmed that the mean biovolume of each species had not changed. After rotifer addition, all rotifers in the samples were enumerated.

### Data Analyses

To determine the relative yield (RY) of polycultures compared to monocultures before rotifer addition, deviation from total yield, D_T_ sensu Loreau [36], was calculated for O_2_ evolution and biovolume. D_T_ is a standardized metric of RY that allows an overall comparison of polyculture productivities across levels of richness. D_T_ was calculated as 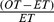 where O_T_ = 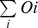 and E_T_ = 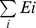 are the observed and expected yields, respectively, of polycultures with i species. Positive values of D_T_ imply overyielding and negative values of D_T_ imply underyielding, relative to the expected productivity of polycultures based on monoculture performance. Compared to other metrics, D_T_ ignores shifts in the numerical dominance of individual species and is used as a rough tool to assess effects of biodiversity [36].

Temporal variability of O_2_ production and total biovolume was used to evaluate the stability of cultures. Stability here refers to constancy of phytoplankton biomass through time [8], [37] and is inversely related to temporal variability. We calculated temporal variability as the coefficient of variation (CV, σ/μ) of O_2_ production and biovolume through time (i.e., for each culture tube, a single CV was calculated from the six sampling points). With these data, we then calculated the mean CV for each of the four richness treatments and the mean CV for each of the 13 species combinations. Therefore, the number of replicates per treatment had no influence on the mean CV calculated. Temporal CV was calculated using transformed values of O_2_ production, as some values were negative towards the end of the experiment. In this paper, we restrict our analyses to experiment-long variability, as the temporal CVs for different periods (i.e. pre-rotifer addition, post-rotifer addition, experiment-long) were similar.

To test for effects of species richness and species combination on net oxygen production, total biovolume, RY and temporal CV we used mixed-model, nested ANOVA, in which species combination (a random factor) was nested within levels of species richness (a fixed factor). Separate ANOVA were conducted for each response variable, and initial biovolume was used as a covariate in the analyses. Because many of the raw data were non-normal, analyses were conducted on log- or rank-transformed data [38], [39]. O_2_ production was not standardized to biomass, as rotifer consumption of O_2_ confounded such standardization. Model diagnostics of residual plots, as suggested by Quinn & Keough [39], were performed for all analyses. To reduce the overall Type I error rate associated with multiple comparisons, the Dunn-Sidak method was used to adjust the α-value for post-hoc Tukey tests [40]. Analyses and model diagnostics were performed in Statistica 10.

To compare the relative importance of species richness and species combination on productivity and stability, we applied the approach suggested by Connolly et al. [41]. Briefly, the importance of species richness (the fixed effect) is defined as the slope of the richness gradient, scaled by the range of richness used in the experiment as 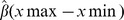. The importance of species combination is defined as the absolute size of the difference between two randomly selected compositions, or 1.128(σ_sc_) where σ_sc_ is the variance component for species combination. For this comparison, the parameters 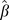 and σ_sc_ were taken directly from the results of the mixed-model analysis (i.e. prediction equation of model and variance component of species combination, respectively).

Finally, the additive partitioning methods of Loreau and Hector [42], using species biovolume, were used to separate the effects of complementarity and selection during the experiment. Positive complementarity, resulting from resource partitioning or positive species interactions, is measured as a positive change in polyculture RY compared to the weighted average of the component species’ monoculture yields. In contrast, positive selection, which occurs when a highly productive species dominates in a polyculture, is indicated by positive covariance between the monoculture yield of a species and its change in RY within the polyculture. Either effect can be positive or negative and the sum of the effects is the net biodiversity effect [42].

## Results

### Productivity and Relative Yield

**Figure 1:**
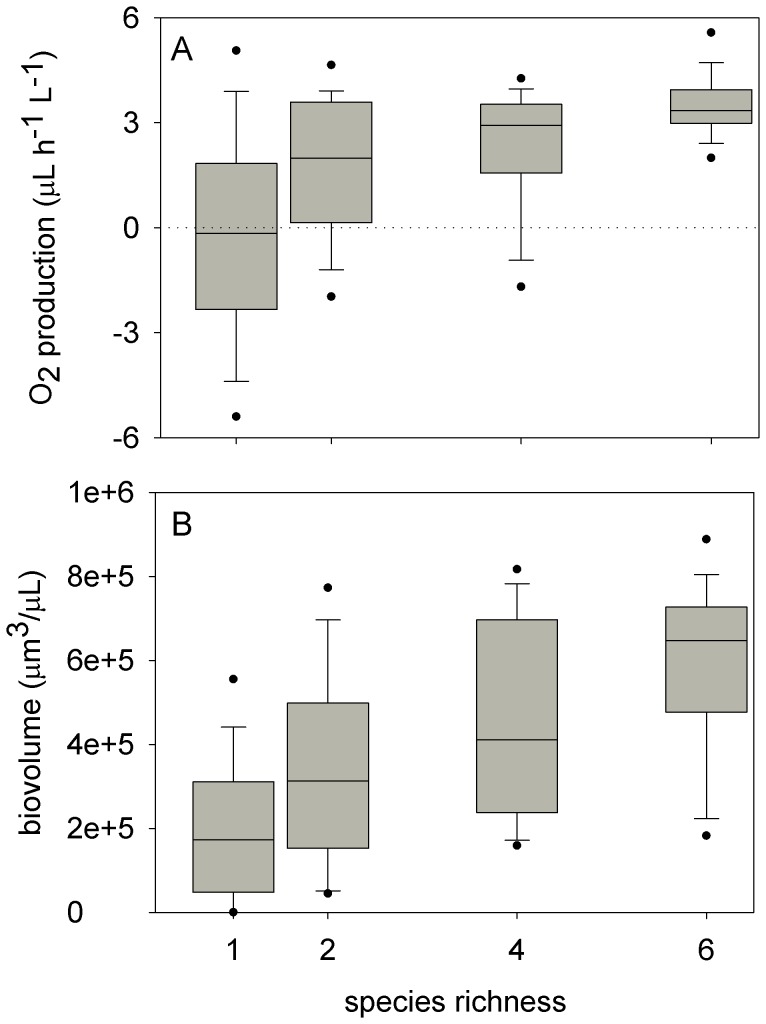
Net oxygen production (A) and total biovolume (B) of phytoplankton monocultures and polycultures. Each box displays the experiment-long median (line within box), 25^th^ and 75^th^ percentiles (box boundaries), 10^th^ and 90^th^ percentiles (lower and upper error bars) and 5^th^ and 95% percentiles (dots). Medians and percentiles were calculated using all data collected throughout the experiment (n = 54 for polycultures, 108 for monocultures). Net oxygen production in the monocultures was centered on zero due to the consumption of oxygen after rotifer addition (see Figure 3A).

**Figure 2:**
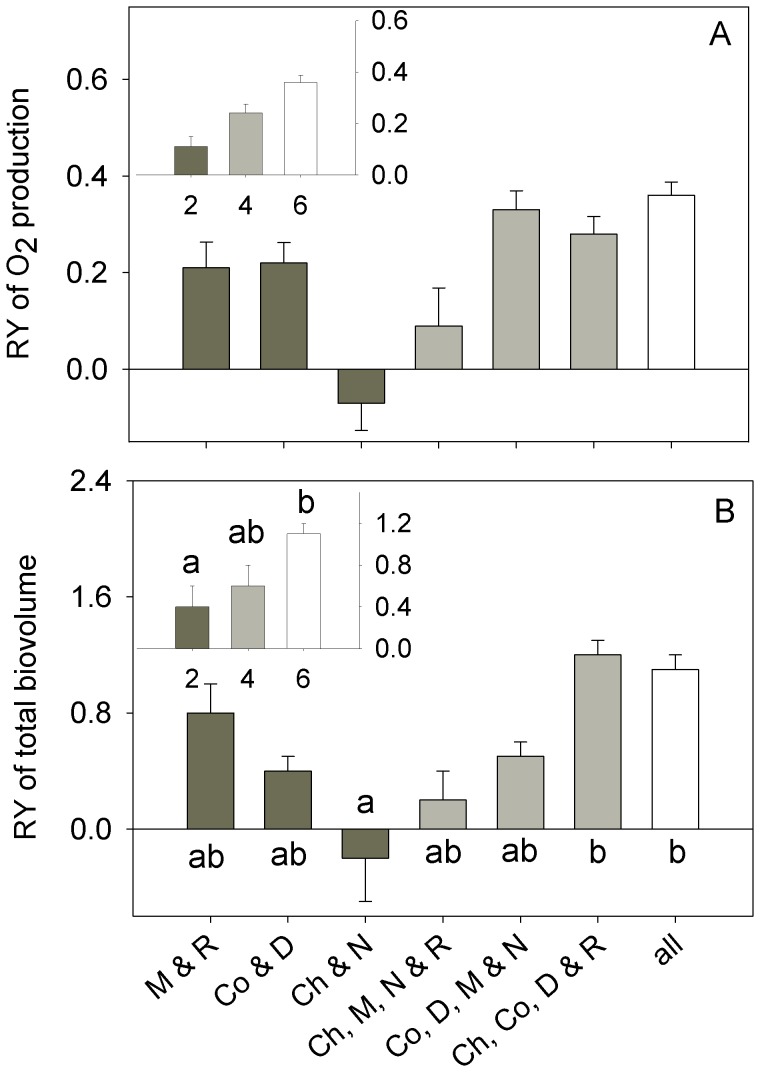
Relative yield of net oxygen production (A) and total biovolume (B). The main plots compare species combinations whereas the insets compare levels of species richness. Bars represent experiment-long means (± SE) calculated for each replicate and sampling day. Letters above each bar indicate the results of the post-hoc Tukey tests associated with ANOVA. Species combinations are listed with each plot, using the following abbreviations: D – *Dunaliella*, N – *Nannochloris*, R – *Rhodomonas*, Ch – *Chaetoceros*, Co – *Coscinodiscus,* M – *Melosira* and all – all species in the six-species combination.

There were significant effects of both species richness and species combination on O_2_ production and total biovolume (Table 3). In general, productivity increased with species richness and the six-species polycultures were the most productive (Fig. 1). However, species combination was approximately four to six times more important than species richness to productivity (Table 3). This strong species combination effect was due to a single, highly productive species: *Dunaliella tertiolecta*. Post-hoc comparisons showed that the biovolume of all polycultures containing *Dunaliella* was significantly higher than that of the other polycultures and monocultures except the *Dunaliella* monoculture (F(9) = 17.84, p<0.0006). Post-hoc comparisons for O_2_ production were similar to those of biovolume (data not shown). Relative Yield (RY) of both O_2_ production and total biovolume before rotifer addition was positive in all polycultures except in the two-species polyculture of *Chaetoceros* and *Nannochloris* (Fig. 2). In the six-species polycultures, RY was highest. There were significant effects of both richness and species combination on the RY of total biovolume, but not on RY of O_2_ production (Table 3, Fig. 2).

**Figure 3:**
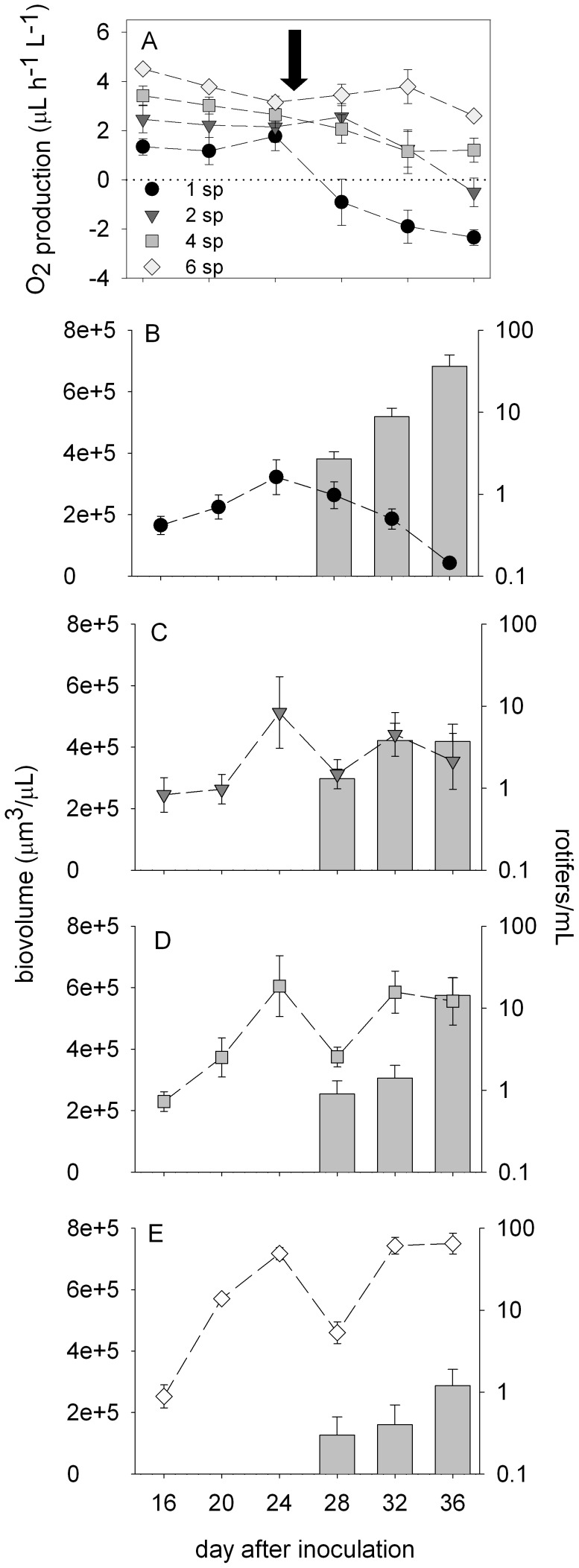
Oxygen production, phytoplankton biovolume and rotifer abundance through time in the different richness treatments. The top figure (A) shows the mean (± SE) net oxygen production of the different richness treatments. The bottom figures show total phytoplankton biovolume (symbols) and rotifer abundance (bars) of the one- (B), two- (C), four- (D) and six-species (e) treatments before rotifer addition (first three points) and after rotifer addition (last three points). Points and bars in b-e represent means ± standard errors (n = nine for polycultures, 18 for monocultures). The arrow indicates the point of rotifer addition at an initial abundance of one rotifer per mL. Rotifer abundance is shown on a log scale.

**Table 3:** Results of mixed model ANOVA comparing the effects of species richness and combination on oxygen production, total phytoplankton biovolume, relative yield (RY), and temporal variability (CV).

response	effect		dominant	ratio
variable	species richness	species combination	variable	
O_2_ production	F(3) = 50.39, **p<0.001**	F(9) = 21.03, **p<0.001**	combination	5.8
	1<2,4<6			
biovolume	F(3) = 70.91, **p<0.001**	F(9) = 17.84, **p<0.001**	combination	4.5
	1<2, 4; 1,2<6			
RY (O_2_ production)	F(2) = 2.58, p = 0.078	F(4) = 1.27, p = 0.285	ns	ns
RY (biovolume)	F(2) = 8.78, **p<0.001**	F(4) = 4.39, **p = 0.002**	combination	2
	2<6			
CV (O_2_ production)	F(3) = 41.88, **p<0.001**	F(9) = 4.60, **p<0.001**	richness	43
	1>2,4,6; 2>6			
CV (biovolume)	F(3) = 10.14, **p<0.001**	F(9) = 4.43, **p<0.001**	richness	18
	1>2,4,6			

Post-hoc Tukey results for effects of richness are expressed in the table as inequalities. See the text for post-hoc comparisons between species combinations. The dominant variable in each ANOVA derived following [41] and the ratio by which it is dominant over the other main factor are listed.

### Stability

Monocultures and polycultures exhibited extremely different responses to rotifer addition. There were short-term negative effects of rotifers on polyculture biomass, but persistent negative effects on monocultures (Fig. 3, S1). The post-rotifer decline in monoculture biomass magnified the pre-rotifer positive richness effects found on O_2_ production and total biovolume. For example, O_2_ production was more similar across treatments before rotifer addition than after rotifer addition. After rotifer addition, values of net O_2_ production diverged, resulting in O_2_ consumption in monocultures (Fig. 3A). Similar divergence was apparent in total biovolume. On the sampling date before rotifer addition, total biovolume varied less than three-fold between monocultures and polycultures (compare across Fig. 3B, C, D, E). In contrast, on the last sampling date, the differences in total biovolume between polycultures and monocultures were orders of magnitude.

**Figure 4:**
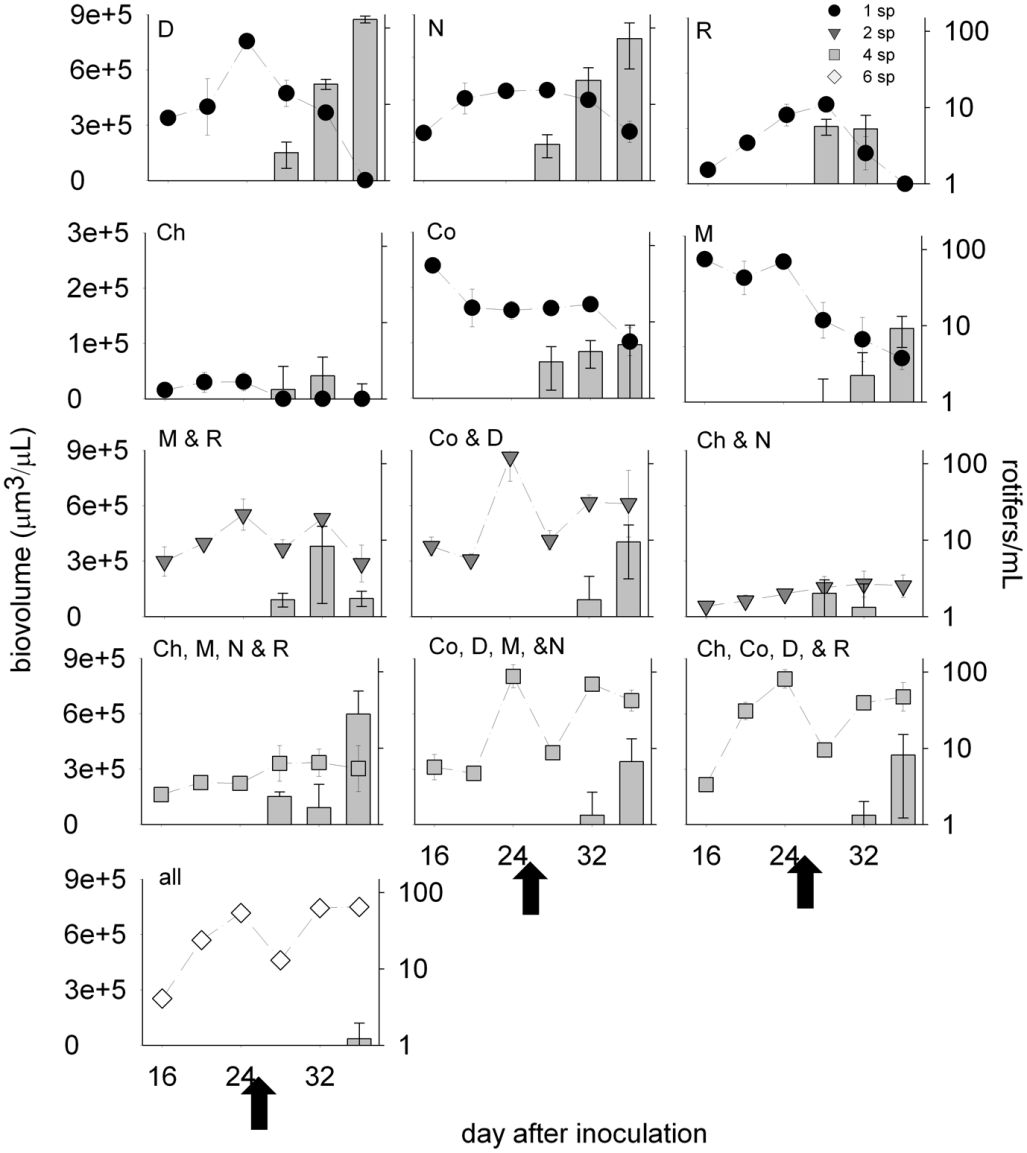
Mean (± SE) phytoplankton biovolume (symbols) and rotifer abundance (bars) of species combinations through time. The arrows indicate the point of rotifer addition at an initial abundance of one rotifer per mL. Species combinations are listed with each plot, using the following abbreviations: D – *Dunaliella*, N – *Nannochloris*, R – *Rhodomonas*, Ch – *Chaetoceros*, Co – *Coscinodiscus,* M – *Melosira,* all – all species in the six-species combination. All primary y-axes are identical with the exception of the y-axis in the row of plots second from the top. Rotifer abundance is shown on a log scale.

Rotifers grazed the monocultures of *Dunaliella*, *Nannochloropsis*, *Rhodomonas* and *Melosira* to or nearly to depletion (Fig. 4). Rotifer abundance was highest and the grazing effects most dramatic in *Dunaliella* monocultures. The monocultures of *Chaetoceros* and *Coscinodiscus* crashed after rotifer addition, but this decline in biomass was not coincident with an increase in rotifer abundance (Fig. 4). Each of the polycultures except the two-species combination of *Chaetoceros* and *Nannochloris* recovered from the negative effects of rotifers (Fig. 4). Rotifer abundance was lowest in the six-species polycultures.

**Figure 5:**
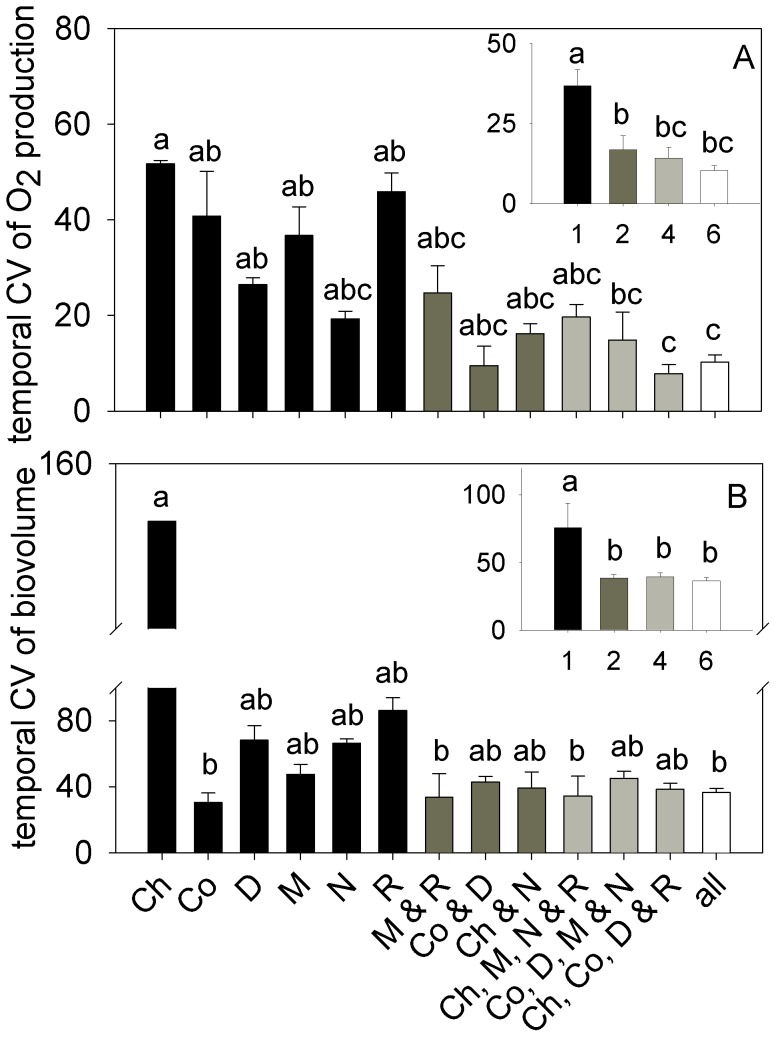
Temporal CV of net oxygen production (A) and total biovolume (B). The main plots compare species combinations whereas the insets compare levels of species richness. Bars represent experiment-long means (± SE) calculated for each replicate and sampling day. Note that the temporal CV was calculated for single culture tubes, such that the mean values for richness treatments and species combinations are not influenced by the number of replicates per treatment. Letters above each bar indicate the results of the post-hoc Tukey tests associated with ANOVA. Species combinations are listed with each plot, using the following abbreviations: D – *Dunaliella*, N – *Nannochloris*, R – *Rhodomonas*, Ch – *Chaetoceros*, Co – *Coscinodiscus,* M – *Melosira* and all – all species in the six-species combination.

Consistent with patterns of species dynamics in polycultures and monocultures, species richness was the dominant variable affecting temporal variability of O_2_ production and total biovolume (Table 3). The effects of species richness were ∼20- to 40-fold greater than the effects of species combination; indeed, O_2_ production and biovolume in the one-species treatments were generally more variable than the two-, four- and six-species richness treatments (Fig. 5). Still, the effects of species combination were also apparent. For example, the temporal CV of *Chaetoceros* biovolume in monoculture was the highest of all coefficients, consistent with its population crash during the experiment (Fig. 4, 5).

### Species Dynamics

Within the polycultures in which *Dunaliella* was present, it accounted for 65–90% of total biovolume and thus dominated patterns of species dynamics (Fig. S2B, C). In polycultures without *Dunaliella*, the smaller species were also consistently dominant: *Rhodomonas* in combination with *Melosira* (Fig. S2A); *Nannochloris* in combination with *Chaetoceros* (data not shown); and *Rhodomonas* in combination with *Chaetoceros*, *Melosira* and *Nannochloris* (data not shown). The biovolume of the larger taxa (i.e. *Chaetoceros, Coscinodiscus* and *Melosira*) was relatively constant before and after rotifer addition (Fig. S2A, B, C), in strong contrast to predictions of the insurance hypothesis based on differences in grazing tolerances. Of the larger cells, *Melosira* and *Coscinodiscus* were the only taxa with measurable biomass in the polycultures before rotifer addition; *Chaetoceros* did not survive in either monoculture or polyculture.

### Complementarity and Selection

Complementarity tended to be positive in all cultures through time, with the exception of the *Chaetoceros* & *Nannochloris* culture, whereas selection was variable in sign, with no discernible pattern throughout the experiment (S3). Complementarity tended to increase with species richness; experiment-long medians of complementarity for total biovolume were 1.7, 2.5 and 2.7 × 10^5^ µm^3^/µL for the two-, four- and six-species treatments, respectively. The *Chaetoceros* & *Nannochloris* treatment exhibited the lowest complementarity compared to other treatments. The median of experiment-long complementarity (1.6 x10^4^ µm^3^/µL) was considerably lower than that of other treatments (1.6 to 3.0 × 10^5^ µm^3^/µL).

## Discussion

In this experiment, we used a diverse pool of taxa to quantify the effects of species combination and richness on productivity and stability of phytoplankton communities. There were strong effects of species combination and richness on productivity and stability, but the relative importance of species combination and richness varied for each of the response variables. Species combination was most important to productivity whereas species richness was most important to stability (Table 3). Before rotifer addition, the most productive cultures were those containing *Dunaliella* (Fig. 4). This finding supports the hypothesis that species-rich communities are productive because they are more likely to contain highly productive species. In contrast, the most species rich communities were the most stable, characterized by low temporal variability in biomass and a recovery after grazer addition. In fact, the polycultures persisted for weeks after rotifer addition (S1). However, the recovery of polycultures was not due to insurance related to grazing tolerances. After rotifer addition, the relative biovolume of the larger cells present in polyculture combinations (*Melosira* and *Coscinodiscus*) did not compensate for the loss of the smaller cells after rotifer addition (Fig. S2). Interestingly, monoculture dynamics indicated that *Melosira* (19–25 µm in diameter) was consumed by rotifers (Fig. 4). These findings are consistent with the ability of rotifers to ingest algae of vastly different size classes and shapes [43], [44], [45]. Although *B. plicatilis* preferentially consumes small particles with an optimal prey size of 8 µm, the approximate size of *Dunaliella*, it can consume particles up to 20–25 µm in diameter, the size of individual *Melosira* cells [46], [47]. Polyculture recovery without compensation by the larger cells therefore points to indirect mechanisms that promoted the temporal stability of polycultures.

We suggest that polyculture recovery was due to reduced rotifer grazing rates in polycultures, resulting in lower rotifer growth rates and a lower net effect of grazing. In particular, the presence of diverse prey in polycultures likely reduced the clearance rates of the rotifers, negatively affecting their growth and allowing polycultures to recover from short-term grazing effects. We define this potential mechanism as the “interference effect” (i.e., different sized algae interfere with the ability of rotifers to adjust their grazing mechanism to one particular prey item). Previous studies have shown that the clearance and growth rate of rotifers, including *B. plicatilis*, varies with prey size [46], [48], [49], morphological characteristics, such as spines [50] and texture [51], and cell abundance [52]. For *B. plicatilis*, relative clearance is highest (100%) on prey of about 8 µm in diameter (the size of *Dunaliella*) and lowest (20%) on prey of 2 µm and 21 µm in diameter (the sizes of *Nannochloris* and *Melosira*, respectively). There is support for selective feeding in *B. plicatilis* [47], [53], which would increase handling time and hence clearance rates in polycultures. In this experiment, rotifers exhibited the highest growth rates in the monocultures of *Dunaliella,* optimally sized prey, and lower growth rates in the monocultures of *Nannochloris* and *Rhodomonas* as well as monocultures of large species, and still lower (negative) growth rates in the six-species polycultures (Fig. 4).

From a predator perspective, it is interesting that resource diversity negatively influenced predator productivity. This finding has been previously documented by Narwani & Mazumder [54], who studied the effects of resource species diversity on the clearance rates of cladoceran zooplankton species. In that study, despite consumer-specific effects of changing resource diversity, resource diversity generally reduced consumer consumption rates. It is important to note that our experimental design employed the use of a single predator species, which has little ecological realism compared to manipulations containing multiple species among trophic levels [55]. For example, with multiple predator species, there may not have been negative effects of phytoplankton diversity on the growth and abundance of rotifers.

The positive complementarity found in this experiment, even before rotifer addition, suggests that either niche partitioning or facilitation may have been important. Although we did not attempt to quantify resource use of the phytoplankton communities, we constructed polycultures from a functionally diverse species pool such that partitioning of light and/or nutrients would be facilitated. Notably, each polyculture consisted of taxa with distinct pigment complements (i.e. chlorophylls *a* and *b* in the chlorophytes, fucoxanthins in the diatoms and phycoerythrins in the cryptophyte). In other studies, positive effects of increasing species richness on phytoplankton productivity have been found when species have varied along functional trait axes, including light requirements [23], [56], [57]. In contrast, when species richness has been manipulated using functionally similar algal taxa, no or weak biodiversity effects on productivity have been found [21], [26]. Our work, which demonstrated positive effects of biodiversity on productivity in diverse phytoplankton polycultures, together with these previous studies, highlights the importance of creating functionally diverse phytoplankton communities to study the mechanisms underlying and effects of biodiversity. Moreover, the effects of biodiversity on ecosystem functioning may depend directly on the range of species’ functional traits. Interestingly, biodiversity effects in this study, using evolutionarily distinct phytoplankton with a range of morphological and biochemical traits, were stronger than the effects found in studies of functionally different vascular plants [58], likely with a narrower trait range.

This work has important implications for understanding how biodiversity losses will affect productivity and stability, particularly in aquatic systems. With respect to phytoplankton communities specifically, potential dominance of harmful algal bloom taxa with climate change may create less productive and stable systems. In addition, it has previously been argued that paradigms of biodiversity differ inherently between terrestrial and aquatic systems, but our work refutes this suggestion. We show that phytoplankton polycultures are more productive and stable than monocultures when exposed to a biological perturbation. We further highlight the importance not only of considering functional groups in exploring the relationships between biodiversity, productivity and stability but also in exploring multi-trophic level interactions in biodiversity experiments.

## Supporting Information

Figure S1 **Photographs showing monocultures and polycultures 24 days after rotifer addition, well after the experiment was terminated.** At this point, there was little measurable biomass in the monocultures and most polycultures consisted of communities dominated by rotifers and *Dunaliella.* These data were not presented in the manuscript because nutrient limitation of phytoplankton growth at this point was certain; however, it is interesting to note that nutrient recycling in the polycultures allowed for sustenance of the dominant organism *Dunaliella*.
(TIF)

Figure S2 **Representative figures showing species dynamics in two- (A), four- (B) and six-species (C) polycultures.**
(TIF)

Figure S3 **Complementarity and selection data for each treatment (species combination/richness level) and sampling date.**
(TXT)
