# Supplementary figures and images for: Biodiversity Increases the Productivity and Stability of Phytoplankton Communities
Source: PLoS One. 2012 Nov 16;7(11):e49397. doi: 10.1371/journal.pone.0049397 (PMC3500281; doi:10.1371/journal.pone.0049397)

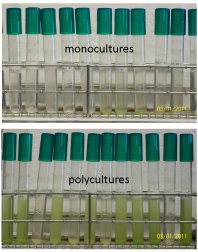

Supplement: Figure S1 — Photographs showing monocultures and polycultures 24 days after rotifer addition, well after the experiment was terminated. At this point, there was little measurable biomass in the monocultures and most polycultures consisted of communities dominated by rotifers and Dunaliella. These data were not presented in the manuscript because nutrient limitation of phytoplankton growth at this point was certain; however, it is interesting to note that nutrient recycling in the polycultures allowed for sustenance of the dominant organism Dunaliella. (TIF) [file pone.0049397.s001.tif]

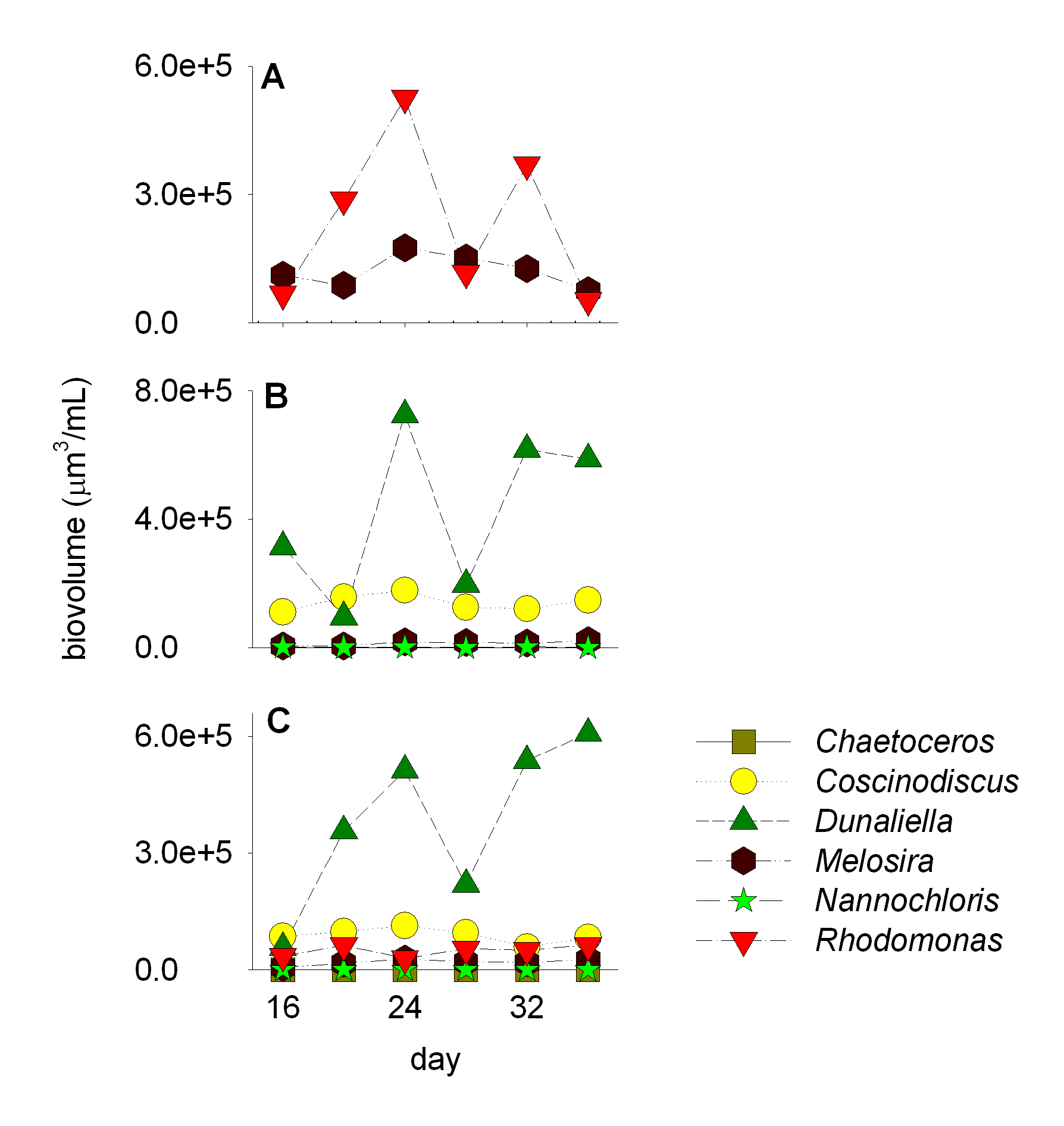

Supplement: Figure S2 — Representative figures showing species dynamics in two- (A), four- (B) and six-species (C) polycultures. (TIF) [file pone.0049397.s002.tif]
